# Parental Challenges in Raising Preschoolers With Attention-Deficit/Hyperactivity Disorder in Mainland China’s First-Tier Cities: Qualitative Study Using Framework Analysis

**DOI:** 10.2196/74047

**Published:** 2025-08-29

**Authors:** Shu-Cheng Chen, Chen-Wen Zhong, Han Li, Xiaoqing Li, Hong-Wang Fung, Lijun Wang, Guo-Tao Wu, Xin-Yue Jiang, Wing-Fai Yeung

**Affiliations:** 1 School of Nursing Hong Kong Polytechnic University Hong Kong China (Hong Kong); 2 The Jockey Club School of Public Health and Primary Care Chinese University of Hong Kong Hong Kong China (Hong Kong); 3 School of Psychology Shenzhen University Shenzhen China; 4 Department of Traditional Chinese Medicine Binzhou Medical University Yantai China; 5 Faculty of Psychology Southwest University Chongqing China; 6 Faculty of Education Southwest University Chongqing China

**Keywords:** attention-deficit/hyperactivity disorder, preschool children, parental challenges, first-tier cities, China, qualitative research

## Abstract

**Background:**

First-tier cities in mainland China present unique challenges for raising children with attention-deficit/hyperactivity disorder (ADHD) due to rapid urbanization, intense academic pressure, and distinct sociocultural dynamics. While existing research has documented ADHD parenting challenges across various contexts, limited attention has been paid to the preschool period in Chinese metropolitan settings, where early intervention is crucial yet complicated by traditional values and evolving health care systems.

**Objective:**

This study aimed to explore the comprehensive challenges faced by parents raising preschoolers with ADHD in these metropolitan contexts.

**Methods:**

We conducted web-based semistructured focus group interviews with 13 parents (n=12, 92% mothers and n=1, 8% fathers; aged between 32 and 44 years) of preschool children with ADHD (n=10, 77% boys and n=3, 23% girls; aged between 5 and 7 years) in first-tier cities in mainland China between March and July 2024. Participants were purposively recruited through the ADHD Mutual Support Alliance’s platform if they had a preschool child with a formal ADHD diagnosis and lived in first-tier cities. Four interviews were conducted (3 focus groups with 4 participants each and 1 pilot individual interview). All interviews were moderated by a practitioner working with populations with ADHD following a semistructured interview guide developed through expert panel consultation. Interviews were audio-recorded and transcribed verbatim. Qualitative data were analyzed using framework analysis, using a theoretical framework approach, with dual-researcher coding and participant verification to ensure methodological rigor. Data were coded and organized manually using Microsoft Word, supported by NVivo software.

**Results:**

Challenges emerged across six major themes: (1) individual psychological and behavioral challenges, including parental emotional burden, self-blame, and difficulties managing ADHD symptoms; (2) microsystem challenges in family and school environment challenges, particularly regarding behavioral management and teacher understanding; (3) mesosystem difficulties in family-school-hospital coordination, highlighted by recurring teacher complaints and inconsistent diagnostic standards; (4) exosystem barriers in work-family balance and health care access, including limited availability of specialized services and financial burdens; (5) macrosystem challenges from societal stigma and traditional educational values, manifesting as discrimination fears and academic pressure; and (6) chronosystem impacts from historical and social changes, notably the COVID-19 pandemic’s influence and gaps in support systems compared to countries with comprehensive health care infrastructure.

**Conclusions:**

Parents face multilayered challenges in raising preschoolers with ADHD, particularly regarding access to specialized health care services, navigation of educational systems, and use of culturally adapted interventions. These challenges are intensified by the unique urban context of intense academic pressure and rapid modernization. Future initiatives should focus on enhancing health care resource accessibility, developing culturally sensitive support programs, implementing systematic educational accommodations, and promoting broader societal awareness of ADHD in mainland China. These findings underscore the necessity for comprehensive ecological interventions that address challenges across all systemic levels while considering the unique characteristics of Chinese metropolitan environments.

## Introduction

### Attention-Deficit/Hyperactivity Disorder

Attention-deficit/hyperactivity disorder (ADHD) is a complex neurodevelopmental disorder characterized by persistent patterns of inattention, hyperactivity, and impulsivity that interfere with functioning or development [1,2]. Globally, approximately 5% of children have ADHD [3], with the pooled worldwide prevalence rate among preschool children estimated at 5.5% (95% CI 3.3%-7.7%) [4]. In China, epidemiological studies indicate that the prevalence of ADHD among children and adolescents was 6.26% (95% CI 5.36%-7.22%) [5], which is comparable to global estimates. The symptoms of ADHD include difficulty sustaining attention, challenges in organizational skills, excessive motor activity, and impulsive behaviors that are developmentally inappropriate and present across multiple settings [6,7]. For preschool children, ADHD manifestations may include marked difficulties in following instructions, completing age-appropriate tasks, and regulating behaviors during structured activities, potentially compromising their early learning experiences and social skill development [8,9]. The impact of ADHD extends beyond the child who is affected, creating significant challenges within the family system, including increased parental stress, strained family relationships, and elevated financial burden associated with health care and educational support [10,11]. Conventional interventions typically encompass a multimodal approach, including behavioral therapy; parent training programs; educational accommodations; and, in some cases, pharmacological treatment, though medication use in preschoolers remains controversial [12,13]. Early identification and intervention are crucial. Untreated childhood ADHD symptoms can precipitate cascading adverse effects on academic, social, and emotional development, establishing patterns that persist into adulthood [14,15]. Timely intervention may mitigate secondary complications and foster adaptive coping strategies, thereby optimizing long-term outcomes [9,16]. Given that the preschool period represents a critical developmental window with heightened neural plasticity and emerging behavioral patterns, targeted support during this stage is paramount for shaping positive developmental trajectories.

### Parental Challenges for Raising Children With ADHD

Previous studies in recent years have explored parental challenges across different cultural contexts when raising children with ADHD. A longitudinal study in the United States documented that mothers of children with ADHD aged between 4 and 12 years experienced significantly higher maternal stress and social isolation [17]. Similarly, in the United Kingdom, a qualitative interview study with 25 mothers of children aged between 5 and 11 revealed substantial program-related barriers with notably high decline rates (68%) [18]. Further evidence from a comparative diary study in the United States reinforced these challenges, tracking 27 mothers of children with ADHD aged between 7 and 11 years who reported markedly higher levels of anger (74.8% vs 51.4%) [19]. A systematic review across Australia, Canada, the United Kingdom, and the United States examining parents of school-aged children (4-18 years) confirmed that mothers consistently faced criticism from teachers and struggled with advocacy while experiencing significant emotional distress and social isolation [20]. In the context of developing nations, a qualitative interview study in South Africa with parents of school-aged children exposed severe burdens, including constant monitoring and emotional distress [21]. In Shantou, China, researchers found unique cultural pressures through interviews with 15 parents of children aged between 7 and 11 years [22]. In addition, a larger cross-sectional study in Sichuan province, China, examining 2497 pupils aged between 6 and 13 years quantified these challenges, revealing that parents of children with ADHD showed a 4.35 times higher risk of depression [23]. While extensive research has documented parental challenges, previous studies primarily focused on school-aged children. There remains a significant gap in understanding these challenges during the crucial preschool period. The preschool period represents a fundamentally different context for several reasons. First, ADHD manifestations in preschoolers are distinct, often characterized by more pronounced hyperactivity and impulsivity, with inattention being less apparent before formal academic demands emerge [8,9]. Second, diagnostic processes are considerably more complex for preschoolers, as distinguishing between age-appropriate developmental variations and clinically significant symptoms requires specialized expertise often unavailable in standard health care settings [12]. Third, intervention approaches differ substantially, with medication being more controversial in younger children and greater emphasis placed on behavioral and environmental modifications [13]. Fourth, parent-educator dynamics in preschool settings involve different expectations and communication patterns compared to primary schools, particularly in China’s distinctive early education system where behavioral conformity is highly valued [24,25]. These fundamental differences necessitate specific investigation of challenges faced by parents of preschoolers with ADHD, especially within China’s unique first-tier urban environments.

### Research Gap

In China, despite the growing attention to childhood ADHD in recent years, systematic investigation of preschoolers remains sparse, even in first-tier cities such as Shenzhen, Beijing, and Shanghai. These economically advanced metropolitan centers present unique challenges through intense academic pressure, rapid urbanization, and distinct sociocultural dynamics, which can be organized into several interconnected factors. First, the high-pressure educational culture creates a fundamental tension for children with ADHD. In the Chinese educational context, *academic burden* refers to the extraordinary pressure placed on children to excel academically from a very young age, characterized by long school hours, extensive homework, frequent high-stakes testing, and numerous extracurricular educational activities. This pressure stems from the deeply entrenched Confucian values that prioritize educational achievement as the primary pathway to success and family honor, combined with intense competition for limited spots in prestigious schools and universities in a population of 1.4 billion. In first-tier cities, this academic pressure is particularly intense due to higher concentrations of educational resources and more affluent, ambitious parents. While recent initiatives such as the “double reduction” policy aim to reduce academic burden [26], the prevalent “tiger parenting” approach continues to emphasize rigorous academic preparation [24]. Second, societal perceptions of behavioral problems create significant stigma and blame for parents [27,28]. Traditional Chinese societal expectations emphasize conformity, self-discipline, and quiet, attentive behavior in children—characteristics that directly conflict with ADHD symptoms. Parents of children with ADHD often face criticism for perceived parental failings, as behavioral issues are commonly attributed to inadequate discipline or moral education rather than neurodevelopmental conditions. Traditional Chinese values prioritizing academic achievement and conformity [29], coupled with fierce competition for early education placement in urban centers [25], significantly intensify parental stress. Moreover, parents have challenges related to societal stigma attributing behavioral issues to inadequate parenting [23]. Third, the intense urban work culture in first-tier cities further compounds these challenges. Parents face unique difficulties related to the demands of high-pressure urban careers [30], with long working hours and intense competitive environments limiting the time and energy available for consistent management of children’s ADHD symptoms. Despite these challenges, parents in first-tier cities have increasingly gained access to professional ADHD information through improved health care resources, mental health education, and international medical perspectives, leading to rising diagnosis rates and earlier interventions [31]. These sociocultural factors create a uniquely challenging environment for parents raising children with ADHD in Chinese first-tier cities. Together, they generate a complex ecosystem where educational demands conflict with children’s neurodevelopmental needs, societal stigma increases psychological burden on parents, and work pressures limit parents’ ability to implement consistent support strategies. Understanding these interconnected factors is essential for developing effective support systems for these families.

To comprehensively understand these multifaceted challenges, the ecological systems theory by Bronfenbrenner [32] provides an ideal theoretical framework. This framework conceptualizes human development and experiences within nested environmental systems: the microsystem (immediate environment and direct interactions, such as family dynamics and parent-child relationships), mesosystem (interconnections between microsystems, such as parent-school collaboration), exosystem (indirect environmental influences, such as health care policies and community resources), macrosystem (broader sociocultural context, including cultural values and societal attitudes toward ADHD), and chronosystem (temporal dimensions and historical changes) [32,33]. This ecological perspective is particularly valuable for examining ADHD-related challenges in the Chinese context because it captures the complex interplay between traditional values, rapid social changes, and family dynamics that uniquely characterize urban Chinese environments. Furthermore, this framework allows us to systematically analyze how various environmental factors at different levels interact to shape parental experiences, moving beyond individual-focused analyses to examine interconnections across personal, institutional, and societal domains [33,34]. The complex challenges faced by parents raising children with ADHD in China’s rapidly urbanizing and highly competitive metropolitan environments warrant an in-depth and comprehensive exploration. This study makes critical contributions beyond existing research in mainland China. While previous studies in Shantou examined school-aged children in smaller cities [22], and research in Sichuan quantitatively assessed parental depression [23], our study uniquely investigated challenges faced by parents of preschoolers within first-tier metropolitan environments. We used the ecological systems framework by Bronfenbrenner [32] to provide a comprehensive analysis of how various environmental systems interact to shape parental experiences, moving beyond the individual-focused analyses of previous studies to examine complex interactions across personal, familial, institutional, and societal domains within China’s most developed urban contexts. Therefore, considering the limited research on parental experiences during the critical preschool period and the unique sociocultural context of Chinese metropolitan areas, we conducted this qualitative study to comprehensively explore and analyze the experiences and challenges encountered by parents raising preschool-aged children with ADHD in first-tier cities in mainland China, providing valuable insights for health care providers, educators, and policymakers in rapidly developing urban contexts.

## Methods

### Study Design

This qualitative study used semistructured focus group discussions to explore parents’ experiences of raising preschool-aged children with ADHD. This method was chosen to facilitate in-depth discussions where participants could share and build upon each other’s perspectives. The COREQ (Consolidated Criteria for Reporting Qualitative Research) checklist was used to guide the reporting of this study [35].

### Participants

All participants, who were parents of preschool children with ADHD, participated in online focus group interviews. Participants were recruited from February to July 2024 through the ADHD Mutual Support Alliance’s WeChat public account, a platform connecting families affected by ADHD across mainland China. The platform provides educational resources and peer support services for parents of children with ADHD. Purposive sampling was used to select participants who could provide rich and diverse perspectives on the research topic [36]. Parents were eligible to participate if they met the following inclusion criteria: (1) having a child aged between 3 and 7 years, (2) the child having received a formal ADHD diagnosis from a qualified health care professional, (3) current residence in first-tier cities (eg, Beijing, Shanghai, Guangzhou, and Shenzhen) in mainland China, (4) ability to communicate in Mandarin, and (5) capability to participate in online interviews via the Tencent Meeting platform. A recruitment advertisement was posted on the platform. Interested parents contacted the research team through the platform. A research assistant conducted online screening interviews with interested parents using a structured questionnaire to determine their eligibility. Those who met all the inclusion criteria were invited to participate in the online focus group interviews. Each focus group discussion was moderated by the principal researcher (GTW), with a research assistant (XYJ) serving as the assistant moderator. All focus group interviews were conducted online via the Tencent Meeting platform to ensure geographical accessibility and convenience for participants from different cities.

### Data Collection

Data collection was conducted through focus group interviews following the practical guidelines by Krueger and Casey [37]. Before implementation, a semistructured interview guide containing 7 open-ended questions (Textbox 1) was developed and subsequently refined through consultations with an expert panel comprising 2 ADHD experts (a researcher with academic expertise in ADHD [GTW] and a practitioner working with populations with ADHD [WFY]) and a qualitative researcher (SCC). The interviews were facilitated by a moderator (GTW), the founder of the ADHD Mutual Support Alliance, who had established a strong rapport with the participants through previous community engagement activities. As an experienced advocate in the ADHD community, the moderator had undergone specialized training in qualitative research methodology under the guidance of an experienced qualitative researcher. The moderator’s responsibilities encompassed establishing session protocols, facilitating group dynamics, and maintaining neutral engagement, while an assistant moderator (XYJ) documented field notes and managed the technical aspects of the Tencent Meeting platform. The moderator’s ADHD expertise enhanced data collection through informed questioning, while having no previous relationship with participants ensured objectivity. Safeguards, including team-reviewed interview guides, regular debriefing sessions, and reflexive journaling, maintained methodological rigor. All sessions were audio-recorded with participants’ written informed consent. To ensure interview quality in the online format, several measures were implemented. All participants received technical orientation before interviews, including guidance on audio settings and connectivity requirements. The assistant moderator monitored technical aspects throughout, addressing disruptions immediately. Participants were encouraged to join from quiet, private locations to minimize distractions. To compensate for reduced nonverbal cues, the moderator used specific online facilitation techniques, including regular verbal check-ins, directed questions to ensure equal participation, and periodic summaries to confirm understanding. All sessions were recorded with redundant audio capture to prevent data loss.

**Semistructured interview guide for focus group discussions with parents of preschool children with attention-deficit/hyperactivity disorder (ADHD) in first-tier Chinese cities.**

- “Could you describe your experience of raising a child with ADHD? Please share your journey.”
- “How do you usually communicate with your child? Do you encounter any difficulties in communication? If so, what are they?”
- “Do you find it challenging to obtain the guidance and support you need? If yes, what are these challenges?”
- “In your opinion, what types of parental care and support are needed for preschool children with ADHD?”
- “What successful experiences have you had in accompanying and guiding your child? (including treatment approaches, etc)”
- “What unsuccessful experiences have you had in accompanying and guiding your child? (including treatment approaches, etc)”
- “What kind of support do you expect from society (schools, hospitals, other organizations, etc) to better help you raise your child?”

### Data Analysis

All interviews were audio-recorded and subsequently transcribed verbatim in Mandarin before analysis. Demographic data were analyzed using descriptive statistics. We conducted framework analysis of the transcripts, guided by ecological systems theory by Bronfenbrenner [32,33], to examine complex social phenomena in their environment. The analysis process followed several systematic steps [38,39]:

1. Familiarization: 2 researchers (GTW and XYJ) independently reviewed all transcripts to achieve data immersion and develop preliminary insights.
2. Identifying the theoretical framework: ecological systems theory by Bronfenbrenner [32] was selected to guide the coding process.
3. Indexing: the researchers independently coded the transcripts, mapping data onto different ecological levels.
4. Charting: data were reorganized into framework matrices, with ecological levels as columns and cases as rows.
5. Mapping and interpretation: themes were identified within and across ecological levels to understand the interconnections between different systemic influences on parental experiences.

To enhance analytic rigor, the 2 researchers conducted independent coding and regularly compared their analyses to resolve discrepancies through discussion. When consensus could not be reached, the principal investigator (GTW) was consulted. The final analytical framework and emerging themes were reviewed by all team members to ensure interpretative validity. The primary processes of data coding and organization were conducted manually in Microsoft Word, with NVivo (Lumivero) software used to facilitate and cross-check coding [40,41].

### Trustworthiness

To establish methodological rigor, we implemented multiple strategies addressing the 4 dimensions of trustworthiness outlined by Lincoln and Guba [42] and further applied in health care research by Forero et al [43]. The study’s credibility was reinforced through a pilot interview that informed subsequent protocol refinement, coupled with regular investigator meetings and dual-researcher coding processes. To ensure dependability, we implemented participant verification of interview transcripts and interpretations while maintaining detailed documentation of our methodological decisions. Participant verification involved email member-checking; participants reviewed interview summaries and gave feedback. The analytical process was strengthened by having a second investigator (SCC) and the principal investigator (GTW) independently verify the coding structure. Confirmability was achieved through participant feedback on interpretations and conscious examination of contradictory cases, while transferability was supported by detailed contextual documentation and theoretical sampling until data saturation [44], with the framework by Bronfenbrenner [32] providing a theoretical foundation for broader application.

### Ethical Considerations

Ethics approval was obtained from the Human Research Committee of the School of Psychology, Shenzhen University (SZU_PSY_2023_068). Written informed consent was obtained from all participants before their participation in the study. To ensure confidentiality, participants were assigned unique identification codes, and all study-related documents and transcripts were deidentified. Audio recordings were securely deleted following transcription completion. To ensure privacy and create a natural, safe atmosphere, participants were instructed to join the interviews from private spaces where no other individuals were present and to disable their video functions during the interviews. To acknowledge their contribution, participants were granted access to a professional development course focused on mindfulness intervention for ADHD, delivered by an expert psychologist specializing in ADHD management.

## Results

### General Characteristics of Data

Three focus group interviews were conducted between March and July 2024 with 13 parents of children with ADHD. Each group included 4 to 5 participants. An additional individual pilot interview was conducted to refine the interview guide and procedures, but its data were not included in the final analysis. Four individuals declined participation due to scheduling conflicts.

### Sample Profile

Thirteen parents (n=12, 92% mothers and n=1, 8% father) participated in the semistructured interviews, conducted across 3 waves following one initial pilot interview. The parents’ ages ranged from 32 to 44 years. All participants had a college education or higher, and most (8/13, 62%) were employed; one mother was a housewife. Their children (n=10, 77% boys and n=3, 23% girls) were aged between 5 and 7 years. ADHD symptoms were first noticed at an average age of 3.2 (SD 2.0) years, and the average duration of treatment was 5.6 (SD 9.0) months. Families reported using a variety of interventions, including medication, cognitive behavioral therapy, and other approaches, either alone or in combination. Detailed demographic characteristics are presented in Table 1.

**Table 1:** Demographic characteristics of parents interviewed about their children with attention-deficit/hyperactivity disorder (ADHD) in first-tier Chinese cities (N=13).

Characteristics			All	Pilot (n=1)	Wave 1 (n=4)	Wave 2 (n=4)	Wave 3 (n=4)
**Child**							
	Age (y), mean (SD; range)		6.3 (0.9; 5-7)	5 (0)	6.5 (0.6; 6-7)	6 (1.2; 5-7)	6.8 (0.5; 6-7)
	**Gender, n (%)**						
		Man	10 (77)	1 (100)	2 (50)	3 (75)	4 (100)
		Woman	3 (23)	0 (0)	2 (50)	1 (25)	0 (0)
	Age at ADHD symptoms (y), mean (SD)		3.2 (2.0)	3 (0)	5.3 (1.7)	2.3 (1.0)	2.3 (1.9)
	Treatment duration (mo), mean (SD)		5.6 (9.0)	0 (0)	4 (5.4)	8.3 (11.3)	6 (12)
**Parents**							
	Age (y), mean (SD; range)		37 (5.0; 33-44)	37 (0)	37.8 (7.4; 33-44)	35.3 (3.6; 32-37)	38 (5; 33-40)
	**Parental role, n (%)**						
		Mother	12 (92)	0 (0)	1 (25)	0 (0)	0 (0)
		Father	1 (8)	1 (100)	3 (75)	4 (100)	4 (100)
	**Level of education, n (%)**						
		College or above	13 (100)	1 (100)	4 (100)	4 (100)	4 (100)
	**Career, n (%)**						
		Employees of public institutions	4 (31)	1 (100)	1 (25)	2 (50)	0 (0)
		Employees of a company	4 (31)	0 (0)	2 (50)	1 (25)	1 (25)
		Freelancers	1 (8)	0 (0)	0 (0)	0 (0)	1 (25)
		Self-employed	1 (8)	0 (0)	0 (0)	0 (0)	1 (25)
		Housewife	1 (8)	0 (0)	0 (0)	1 (25)	0 (0)
		Others	2 (15)	0 (0)	1 (25)	0 (0)	1 (25)
	**Monthly family income (**¥**), n (%)**						
		≤10,000 (US $1400)	1 (8)	0 (0)	1 (25)	0 (0)	0 (0)
		10,001-29,999 (US $1401-4200)	7 (54)	1 (100)	2 (50)	2 (50)	2 (50)
		30,000-49,999 (US $4201-7000)	2 (15)	0 (0)	1 (25)	0 (0)	1 (25)
		≥50,000 (US $7001)	3 (23)	0 (0)	0 (0)	2 (50)	1 (25)

### Major Themes

#### Overview

**Figure 1:**
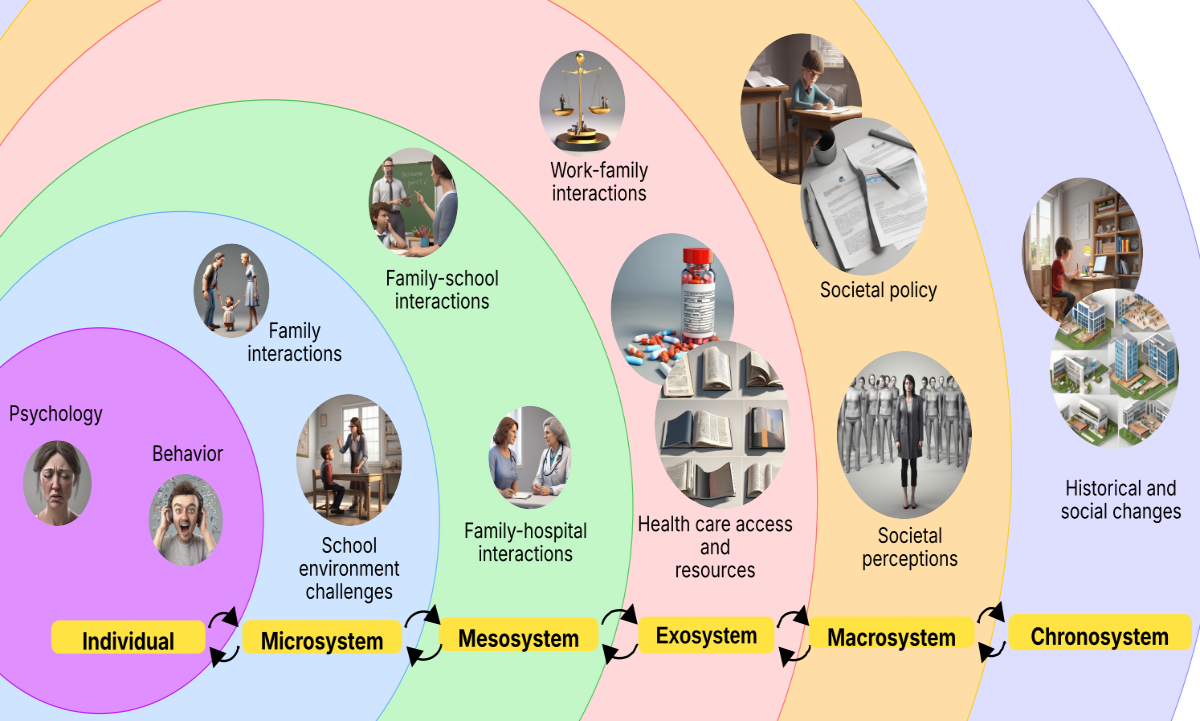
Bronfenbrenner's ecological systems framework applied to parental challenges in raising pre-schoolers with attention-deficit/hyperactivity disorder (ADHD) in first-tier cities of mainland China. This figure depicts the nested environmental systems influencing preschool children with ADHD and their families in Chinese first-tier cities. Based on Bronfenbrenner's ecological systems theory, it illustrates six interconnected levels: individual (psychological and behavioral factors), microsystem (immediate family and school environments), mesosystem (family-school-hospital interactions), exosystem (work-family balance and health care access), macrosystem (societal policies and perceptions), and chronosystem (historical and social changes). Icons represent themes from focus group interviews (N=13) conducted in 2024, highlighting challenges unique to China's urban context of intense academic pressure and evolving health care systems.

Through a thorough analysis of the transcripts and participant quotations, six themes emerged aligned with the ecological framework by Bronfenbrenner [32]: (1) individual level, encompassing psychological factors and behavioral factors; (2) microsystem, involving family interactions and school environment challenges; (3) mesosystem, including family-school interactions and family-hospital interactions; (4) exosystem, covering work-family interactions and health care access issues; (5) macrosystem, reflecting societal policy and societal perceptions; and (6) chronosystem, capturing historical and social changes. Figure 1 [32] illustrates the ecological systems framework by Bronfenbrenner [32] as applied to parental challenges. These themes, their respective subthemes, and supporting participant quotations are detailed in Multimedia Appendix 1.

#### Individual

##### Overview

At the individual level, parents of preschool children with ADHD experienced significant psychological and behavioral challenges. Parents experienced persistent psychological strain, manifesting as emotional distress, internalized self-blame, and anxiety regarding their parenting efficacy. The demands of managing their child’s ADHD symptoms contributed to feelings of exhaustion and ongoing concern for their child’s future development. Some parents also described challenges related to their own behavioral characteristics, including the impact of potential parental ADHD traits on family functioning. Concerns about children’s development extended to worries about social adaptation, academic readiness, and emotional regulation, intensifying parental stress.

##### Psychological Factors

The psychological factors manifested in 2 primary ways. First, parents reported intense emotional challenges, including feelings of anger, frustration, self-blame, depression, and anxiety. One mother described her emotional struggle as follows:

Sometimes I can’t control myself. When I’m in a bad mood, I feel really guilty. When my child misbehaves, I might not handle it well, and then I blame myself. For instance, when I punish him by spanking, I feel terrible inside afterwards.

Participant 09

Second, parents faced substantial parenting burdens, particularly regarding time demands and physical and emotional energy depletion. One parent expressed this overwhelming burden as follows:

I wish I could be like Nezha with three heads and six arms, because I feel I’m already doing everything I possibly can. I’ve given her all my available time when she’s at home, but there are still things I can’t manage. For example, I can’t cook and play with her at the same time, right?

Participant 04

##### Behavioral Factors

In terms of behavioral factors, parents faced 2 main challenges. Parents usually dealt with their own behavioral issues, including ADHD symptoms and oppositional behavior. One parent shared their personal struggle as follows:

I’m diagnosed with ADHD myself...My personality is confrontational, hyperactive, and impulsive—I have the combined type. All the typical emotional issues, including depression—I’ve got them all.

Participant 12

In addition, parents expressed significant concerns about their children’s development, particularly regarding social skills, academic performance, and emotional regulation. One parent articulated as follows:

I worry that the more time he spends immersed in (animated videos), the less he’ll want to do things that require calm thinking or interact with others...I’m also concerned about his emotional issues. When he’s unhappy about something, he might just walk away without a word and completely give up on his tasks. I worry that he’ll have poor self-control when he grows up.

Participant 09

#### Microsystem

##### Overview

At the microsystem level, challenges in 2 primary domains emerged from the analysis: family interactions and school environment challenges. Within the immediate family context, parents reported difficulties in maintaining consistent behavioral management strategies and achieving family cohesion. Conflicting approaches among caregivers and communication barriers within the family frequently led to strained relationships and limited support. In the preschool environment, parents identified insufficient teacher awareness and understanding of ADHD, resulting in limited accommodation of their children’s needs and increased behavioral challenges at school.

##### Family Interactions

Within family interactions, parents identified significant challenges in managing their children’s behaviors and maintaining family harmony. Parents acknowledged their difficulties in providing consistent and timely intervention for their children’s problematic behaviors, such as procrastination, distraction, and impulsivity. One parent reflected on how their permissive approach at home translated into behavioral issues at school:

Take singing in the bathroom—when he sang or yelled loudly at home, I didn’t correct him right away. So at school, he thinks it’s fine to yell or sing in the bathroom...And about being disorganized—I never insisted on him keeping things organized at home, so naturally at school he’s careless with his stuff and often forgets his homework.

Participant 02

Family conflicts emerged as another significant challenge, characterized by negative emotions, communication barriers, and inconsistent parenting approaches among family members. The complexity of multicaregiver dynamics was highlighted by 1 parent:

Since three adults (me, my husband, and my mom) are raising the child together, there are lots of differing opinions and we all have short tempers, so the child endures a lot of emotional stress.

Participant 11

##### School Environment Challenges

In terms of school environment challenges, parents reported challenges stemming from educators’ limited understanding of ADHD. This lack of awareness affected how teachers perceived and responded to their children’s behaviors. One parent noted the following:

I think teachers—from preschool through elementary and middle school—they don’t really understand this condition (ADHD). They just think the child is strange.

Participant 03

#### Mesosystem

##### Overview

At the mesosystem level, the intersection of family, school, and health care systems generated additional stress for parents. Interactions with teachers were often characterized by recurring reports of problematic behavior and challenges in constructive communication. Parents also highlighted difficulties coordinating with health care professionals due to inconsistent diagnostic criteria and fragmented service provision, which contributed to confusion regarding the appropriate course of action and uncertainty about their child’s prognosis and intervention pathways.

##### Family-School Interactions

In the context of family-school interactions, parents experienced significant stress from recurring teacher complaints about their children’s behavior. The emotional toll of these interactions was vividly described by 1 parent as follows:

His kindergarten teacher frequently reported that he wouldn’t follow instructions. She had to remind him about 5-6 times during one class, which was really excessive. And after each reminder, it wouldn’t even last two or three minutes before he was off-task again.

Participant 09

Furthermore, parents struggled to effectively manage their children’s negative attitudes following teacher criticism. One parent’s reflective account highlighted the counterproductive nature of their initial approach:

When teachers reported issues to me, I immediately scolded him: ‘How could you do this? Why did you make this mistake again after we’ve discussed it?’ Looking back, my approach was wrong. He was already criticized at school, then came home to face more criticism from me siding with the teacher. This confrontational communication eventually led him to stop listening to me.

Participant 08

##### Family-Hospital Interactions

Regarding family-hospital interactions, parents encountered challenges on 2 fronts. First, many parents expressed doubts about the reliability or accuracy of the ADHD diagnosis due to inconsistent diagnostic procedures and standards across different hospitals and clinicians. One parent observed the following:

When we later saw another doctor, they didn’t conduct an IQ test. These tests are now administered by nurses across different hospitals, and the assessment quality varies significantly between experienced and inexperienced staff.

Participant 08

Second, parents frequently reported feeling lost and overwhelmed when navigating fragmented and complex health care pathways, unsure about next steps for intervention and support. One parent noted the following:

In the process of raising him, I would start reading books about ADHD and sign up for online courses, but I often abandoned these efforts halfway due to work commitments and other reasons. This pattern kept repeating itself in a continuous cycle.

Participant 14

These forms of confusion and uncertainty led to increased stress and hesitancy in decision-making about their child’s care.

#### Exosystem

##### Overview

External circumstances, particularly related to work and health care access, further complicated the parenting experience. Parents struggled to balance demanding professional responsibilities with the intensive needs of their children, frequently facing constraints in time, energy, and resources. Barriers to accessing specialized health care services—including high costs, limited availability, and lack of individualized or culturally relevant interventions—were repeatedly emphasized. These factors collectively exacerbated the burden on families.

##### Work-Family Interactions

In the domain of work-family interactions, parents faced substantial challenges in balancing professional responsibilities with their children’s needs. One parent described how work commitments affected early childcare:

After my child turned one year, I worked full-time. Until age three, the grandmother took care of them while I rarely came home, usually caught up in my company work. I was somewhat lacking in childcare during this period.

Participant 12

Some parents made significant career sacrifices to accommodate their children’s needs, as illustrated by another parent’s experience:

Due to my child’s ADHD, I quit my job last year. I decided to prioritize helping my child before focusing on my career, believing that career success means little if my child isn’t doing well. This decision had a significant impact on my life.

Participant 08

##### Health Care Access and Resources

The health care access and resources domain presented multiple challenges for families. The financial burden of ADHD interventions emerged as a significant concern; 1 parent detailed the following:

Currently, we’re doing sensory integration training and medication—all self-funded as insurance doesn’t cover these. The training is extremely expensive at nearly 300 yuan per session, 2-3 times weekly. With desensitization therapy on top of that, the financial burden is heavy.

Participant 10

Access to professional medical resources proved particularly challenging, especially at prestigious institutions. A parent described their frustration as follows:

Getting an appointment at Peking University’s 6th Hospital (China’s most authoritative hospital for child mental health) for pediatric services is incredibly difficult. The slots disappear within seconds—it’s practically impossible to get one. I haven’t succeeded even once. I wonder why these pediatric appointments are in such high demand and who manages to get them? It’s puzzling how they vanish instantly.

Participant 03

Parents also highlighted the lack of personalized medical services. One parent, who had personal experience with ADHD, observed the following:

I have ADHD myself, and I’ve noticed many differences between my symptoms and my son’s. The biggest challenge is getting personalized treatment. Take West China Hospital in Chengdu (one of the best hospital in Southwest China) for example—there are only one or two truly respected specialists. Not everyone responds well to the same treatment approach. Even nationwide, I feel there are very few doctors who can provide truly individualized treatment.

Participant 17

In addition, parents struggled to find culturally relevant parenting resources. One parent explained the following:

During the kindergarten stage, we want to provide individualized parenting at home. While there are many parenting books on the market, most are written by and for Westerners, with very few by Chinese authors. Given the unique Chinese educational environment, many Western parenting methods simply aren’t applicable here.

Participant 04

#### Macrosystem

##### Overview

External circumstances, particularly related to work and health care access, further complicated the parenting experience. Parents struggled to balance demanding professional responsibilities with the intensive needs of their children, frequently facing constraints in time, energy, and resources. Barriers to accessing specialized health care services—including high costs, limited availability, and lack of individualized or culturally relevant interventions—were repeatedly emphasized. These factors collectively exacerbated the burden on families.

##### Societal Policy

Regarding societal policy, parents expressed deep frustration about the lack of comprehensive support systems. One parent articulated their pessimism about systemic change:

I couldn’t see significant progress happening in China over the next five to ten years. While small groups are working hard to push for change, their progress is inevitably slow. It’s not that we aren’t trying, but real change requires support from higher policy levels. Even when research institutions achieve results, without sufficient backing, it’s all talk. This situation leaves me feeling somewhat helpless.

Participant 12

##### Societal Perceptions

In terms of societal perceptions, parents encountered multiple challenges related to stigmatization and traditional educational values. The fear of social discrimination deeply affected parents’ willingness to disclose their children’s condition; 1 parent revealed the following:

It’s hard for me to admit to other parents that my child has ADHD. I’m afraid they’ll go home and tell their children to stay away from my child, warning them “be careful, he might hit you.” They’ll become defensive and prejudiced. This social stigma makes me feel powerless.

Participant 16

The broader issue of societal tolerance was highlighted by another parent:

These days, it’s not just parents of ADHD or autistic children—once you become a mother, you’re automatically downgraded to a second-class citizen. Society’s tolerance needs improvement, not just for special needs children, but for all children in general.

Participant 11

The severity of social stigma was further illustrated through a disturbing example:

We saw news about a child with ADHD whose entire class—over 40 parents—joined forces to demand their transfer to another school. If this happened to my child, it would feel terribly unfair. Yet, I can somehow understand those parents’ perspective too.

Participant 10

The pressure of traditional educational values created additional challenges. One parent emotionally described their fears about their child’s academic future:

My child barely passes each subject. In Beijing, this likely means they won’t get into high school. Their poor short-term memory means they’ll gradually fall further behind their peers. Yet they still have to attend school with children their age. It’s terrifying...unless they quit school entirely, but...(sobbing).

Participant 03

The systemic constraints on teachers were also acknowledged:

Teachers have their own evaluation system. When they’re being assessed based on academic performance, how can they spare extra energy for children with special needs?

Participant 17

#### Chronosystem

##### Overview

The chronosystem-level analysis highlighted how temporal changes, both societal and historical, influenced the experiences of families managing ADHD. While our study design is cross-sectional, parents’ narratives included important reflections on temporal and historical factors that influence their experiences. These chronosystem-related findings represent parents’ retrospective perceptions and comparisons rather than longitudinal observations of historical changes. Temporal and historical factors shaped parental experiences, with recent societal changes and events, such as the COVID-19 pandemic, influencing family dynamics and access to support. Parents frequently compared the current environment with perceived support systems in other countries, highlighting persistent gaps in service provision and understanding of ADHD over time. While these reflections were based on parental perceptions rather than systematic longitudinal analysis, they nonetheless provided important context for understanding the evolving challenges faced by families.

##### Historical and Social Changes

The COVID-19 pandemic emerged as a significant temporal factor that exacerbated ADHD symptoms in children. One parent reflected on how pandemic-related restrictions potentially contributed to their child’s condition:

His ADHD might have developed during the pandemic due to lack of exercise and my frequent business trips—sometimes I’d get stuck somewhere due to lockdowns. I wasn’t there enough for him...At first, I just thought he was being naughty, but after the pandemic, when he returned to kindergarten, he started ignoring teachers’ instructions.

Participant 02

The ongoing evolution of ADHD support systems in China represented another crucial chronosystemic element. Parents often compared their experiences with more developed support systems in other countries, highlighting the current gaps in China’s approach. One parent drew a compelling comparison:

In some countries, once a child is diagnosed with ADHD, they’re provided with a service dog at school. When the dog sits quietly, the child sits too, and others don’t view them strangely. Our country still has a way to go in this regard—there’s a gap in understanding. Teachers might not even understand ADHD and try to enforce standard expectations, causing more pressure and psychological trauma for these children.

Participant 09

This sentiment was echoed by another parent who succinctly captured the developmental gap in support systems:

There’s still a significant gap between us and some developed countries. Our supportive systems are not adequate yet.

Participant 07

While parents perceived the COVID-19 pandemic and international disparities as influential, our cross-sectional design precludes causal attribution. These accounts should be interpreted as illustrative examples of chronosystem influences rather than systematically analyzed or central findings.

## Discussion

### Principal Findings

This is the first study to comprehensively examine the challenges faced by parents raising preschoolers with ADHD in Chinese first-tier cities, revealing complex interconnections across multiple systemic levels. Our findings demonstrate that parental experiences are shaped by intricate interactions between individual psychological burdens, immediate family dynamics, institutional relationships, societal structures, and temporal changes. At the individual level, parents experienced significant emotional challenges and behavioral management difficulties, compounded by their own potential ADHD symptoms. The microsystem analysis revealed substantial strain in both family relationships and school environment challenges, while the mesosystem highlighted particular challenges in navigating between educational and health care institutions. Within the exosystem, parents faced significant work-life balance challenges and encountered substantial barriers in accessing appropriate health care resources, including financial burdens and limited availability of specialized services. The macrosystem analysis exposed how traditional Chinese educational values and societal stigma profoundly impact parenting experiences, and our analysis of chronosystem elements revealed how parents perceived recent events, particularly the COVID-19 pandemic, and international disparities in ADHD support systems as influential factors. However, these references were anecdotal and based on parental perceptions. While parents perceived the COVID-19 pandemic and international disparities as influential, our cross-sectional design precludes causal attribution. Therefore, these findings should be interpreted as illustrative examples of chronosystem influences, rather than systematically analyzed or central conclusions. This study specifically examined these challenges within the context of Chinese first-tier cities, where rapid urbanization, intense academic pressure, and evolving health care systems create a unique environment for families managing preschool ADHD. The application of the ecological framework by Bronfenbrenner [32] provided valuable insights into how these various systemic levels interact and influence parental experiences, highlighting the need for comprehensive, multilevel support systems.

This study’s application of the ecological framework by Bronfenbrenner [32] to examine ADHD-related challenges in Chinese first-tier cities advances beyond previous research. Our ecological analysis revealed complex influences between multiple system levels that shape parental experiences. Particularly notable is our finding regarding the paradoxical relationship between improved diagnostic capabilities and inadequate intervention resources in first-tier cities. Our focus on preschoolers revealed distinctive early-stage challenges, including early identification difficulties, accessing appropriate interventions, and managing the critical transition to educational settings during a foundational developmental period.

### Comparisons With Previous Studies

Several findings from our study align with previous research in various cultural contexts. The significant emotional burden and psychological distress experienced by parents, particularly mothers, echo findings from Western studies. For instance, our participants’ reports of anxiety, depression, and self-blame parallel those documented in a US longitudinal study where mothers of children with ADHD experienced significantly higher maternal stress and social isolation [10]. The challenges in family-school interactions and recurring teacher complaints identified in our study reflect similar findings from a UK qualitative study, where mothers faced substantial criticism from educators [45]. In addition, our findings regarding social stigma and discrimination align with research from multiple cultural contexts, where parents reported severe emotional distress and social isolation [46]. The financial burden and health care access difficulties reported by our participants mirror findings from previous Chinese studies, particularly research that highlighted unique cultural pressures on parents [47]. The impact of work-family conflicts on parenting quality corresponds with findings from a systematic review across Western countries, where mothers consistently struggled to balance career demands with caregiving responsibilities [48]. Furthermore, our participants’ experiences with insufficient institutional support systems align with findings from other Asian contexts, where parents of children with ADHD showed significantly higher risks of depression due to inadequate support structures [49]. These consistencies across different cultural contexts suggest some universal challenges in raising children with ADHD, despite varying sociocultural backgrounds.

Several of our findings diverge notably from previous research literature. While Western studies have consistently reported teachers as primary sources of support and key collaborators in ADHD management [50], our participants encountered significant challenges with school personnel, characterized by frequent conflicts, misunderstandings, and limited support. Teachers in our study were often described as resistant to accommodating these students’ needs, possibly reflecting the intense academic pressures and rigid behavioral expectations in the Chinese education system. Perhaps most strikingly, while previous studies have emphasized the crucial role of extended family support in Chinese contexts [51], our participants reported that extended family members often became sources of additional stress rather than support, primarily due to conflicting views about ADHD management and disagreements over treatment approaches. This difference in teacher support may be attributed to several factors unique to the Chinese educational context: (1) the intense performance-oriented evaluation system that prioritizes academic achievement over behavioral accommodation, (2) significant knowledge gaps in ADHD training within Chinese teacher education programs, (3) larger class sizes in Chinese schools (typically 40-50 students) that limit individualized attention, and (4) the cultural emphasis on conformity and discipline that may frame ADHD behaviors as willful misconduct rather than neurodevelopmental differences. These structural and cultural factors create an environment where teachers face institutional disincentives for accommodating children with ADHD, despite their potential personal willingness to support these students. This may relate to the fact that preschool teachers in China are generally trained to emphasize play-based learning and social-emotional development, whereas primary school teachers are oriented toward academic achievement, standardized curricula, and classroom discipline. These differing expectations may shape how teachers respond to children with ADHD [52].

Our findings reveal a complex interplay of systemic challenges in China’s ADHD health care system. First, we uncovered an unprecedented severity in accessing specialized ADHD services that substantially exceeds previously documented health care barriers. While earlier studies noted general appointment difficulties [53], our findings demonstrate an extraordinary level of access constraints, with participants reporting that securing appointments at leading institutions has become virtually impossible—slots are filled within seconds of release. A second critical dimension emerges in the paradoxical relationship between diagnostic capabilities and treatment delivery. Despite significant improvements in ADHD awareness and diagnostic rates in first-tier cities [54], there exists a substantial gap in individualized, culturally appropriate interventions. Our analysis reveals that while practitioners can readily identify ADHD cases, their therapeutic approaches lack necessary customization, predominantly relying on direct translations of Western materials and standardized treatment regimens that fail to account for China’s unique sociocultural context [55]. This standardization extends to both clinical interventions and parent training resources, which inadequately address Chinese family dynamics and cultural values around child development [56]. The situation is further compounded by China’s intensive, exam-oriented education system, where academic performance metrics overshadow behavioral support needs, creating what participants described as an “impossible balance” between managing symptoms and meeting rigorous academic standards. The absence of references to alternative educational options in our data reflects the significant infrastructural limitations even in mainland China’s most developed urban centers [57,58]. Despite the advanced economic development of first-tier cities, specialized educational pathways for preschoolers with ADHD remain virtually nonexistent within the mainstream system [59]. Unlike some Western contexts [60], mainland China’s educational approach offers minimal formalized alternatives at the preschool level, with most children attending standard kindergartens regardless of neurodevelopmental needs [52,61]. The few specialized institutions that exist primarily operate in the private sector, are often financially inaccessible to average families, and vary considerably in quality due to limited regulatory standards [62]. This educational landscape, when combined with the diagnostic inconsistencies noted by participants, creates a particularly challenging situation where parents may obtain a diagnosis but find no specialized educational pathways designed to accommodate their children’s needs within the conventional system.

### Implications

Our findings have critical implications for multiple stakeholders in first-tier Chinese cities’ ADHD ecosystem. For health care administrators in major metropolitan hospitals, these results highlight the necessity of optimizing current ADHD specialist resources, particularly given the high diagnosed patient volumes in urban areas. Mental health professionals and researchers in leading institutions should prioritize developing standardized yet flexible intervention protocols that address the unique challenges faced by urban Chinese families, such as high academic pressure and limited parent-child interaction time. For public health educators and community organizations in these developed urban areas, efforts should focus on enhancing workplace- and school-based ADHD awareness programs, particularly targeting high-achieving professionals and educators who may overlook ADHD symptoms due to academic or career success. Educational administrators in key urban schools should develop practical accommodation policies that balance the rigorous academic standards of first-tier cities with the support needs of students with ADHD. Importantly, such systematic educational accommodations should be designed to align with the objectives of China’s “*double reduction*” policy, supporting the reduction of students’ academic burden while meeting the diverse needs of learners with ADHD. In addition, medical education institutions in these metropolitan areas should expand their ADHD-specific training programs to meet the growing demand for qualified professionals in urban settings.

The notable absence of school principals in parents’ accounts reflects the distinctive hierarchical structure of Chinese educational institutions, where administrative leadership remains relatively distant from daily parent interactions [63,64]. This contrasts with Western educational models, where principals may play more visible roles in special education support systems [65]. At the preschool level in Chinese first-tier cities, our findings suggest that teachers function as institutional gatekeepers, mediating between families and higher administrative levels [66]. This hierarchical distance may inadvertently limit opportunities for systemic policy implementation supporting children with ADHD, as principals, who typically possess greater authority to implement accommodations [67], remain removed from direct awareness of these children’s needs. Compared to Western contexts, where collaborative models between teachers and mental health professionals are more common, Chinese teachers often exhibit resistance, partly due to systemic barriers, such as comparatively larger class sizes [68]. Therefore, future research should specifically examine the role of school leadership in supporting preschool children with ADHD through targeted interviews with both principals and parents who have had significant administrative interactions.

### Limitations

Several limitations of this study warrant consideration. First, the participant demographics pose potential generalizability limitations, as our sample (12/13, 92% mothers and 1/13, 8% father) was overwhelmingly composed of highly educated mothers (100% college educated). This homogeneity means that our findings may not fully represent the experiences of families from lower socioeconomic backgrounds or those of fathers in first-tier Chinese cities. While this reflects broader patterns in these urban centers, where highly educated parents often demonstrate greater awareness of developmental concerns [69] and maintain higher academic expectations for their children [70], and are more proactive in developmental monitoring and information-seeking [71], families with less education or fewer resources may encounter different or additional barriers that are not captured in our study. Therefore, the transferability of our findings is limited. Future studies should purposefully recruit more fathers and families from diverse socioeconomic backgrounds to better capture the full range of parental experiences in urban China. Second, while our research interview guide included questions about parenting strategies, this manuscript deliberately focused on challenges, as understanding these fundamental obstacles is crucial for developing effective support systems and interventions. The valuable insights regarding parenting strategies and coping mechanisms, though important, will be reported in the future to allow for comprehensive exploration of each aspect. In addition, while our results include chronosystem-related findings, our cross-sectional design limits conclusions about historical changes over time. These findings represent parents’ retrospective perceptions and comparisons rather than systematic longitudinal data. Finally, our study relied solely on parental perspectives, and future research would benefit from incorporating multiple stakeholders’ viewpoints, including children, teachers, and health care providers, to provide a more holistic understanding of the ADHD care ecosystem in urban China.

### Conclusions

Our findings illuminate the multilayered challenges confronting parents of preschoolers with ADHD in first-tier Chinese cities, encompassing individual psychological burdens, health care access barriers, and sociocultural constraints. Parents face substantial obstacles in accessing specialized services, navigating educational stigma, and using culturally appropriate interventions, challenges intensified by the unique urban context of intense academic pressure and rapid modernization. These challenges underscore the necessity for comprehensive ecological interventions, encompassing enhanced health care accessibility, culturally sensitive support programs, systematic educational accommodations, and broader societal awareness initiatives for ADHD.

## Appendix

Multimedia Appendix 1 Thematic coding framework of parental challenges in raising preschool children with attention-deficit/hyperactivity disorder in first-tier Chinese cities.

## Abbreviations

ADHD: attention-deficit/hyperactivity disorder
COREQ: Consolidated Criteria for Reporting Qualitative Research

## Data Availability

The datasets generated or analyzed during this study are available from the corresponding author on reasonable request.
