# Supplementary material for: Parental Challenges in Raising Preschoolers With Attention-Deficit/Hyperactivity Disorder in Mainland China’s First-Tier Cities: Qualitative Study Using Framework Analysis
Source: JMIR Form Res. 2025 Aug 29;9:e74047. doi: 10.2196/74047 (PMC12432475; doi:10.2196/74047)
Supplement: Multimedia Appendix 1 [file formative_v9i1e74047_app1.docx]

**Appendix 1. Thematic coding framework of parental challenges in raising preschool children with attention-deficit/hyperactivity disorder (ADHD) in first-tier Chinese cities.**

| Ecological level and theme | | | Representative quote |
| --- | --- | --- | --- |
| **Individual** | | | |
|  | **Psychological factors** | | |
|  |  | Parental emotional challenges (eg, anger, frustration, self-blame, depression, and anxiety) | - “Sometimes I can’t control myself. When I’m in a bad mood, I feel really guilty. When my child misbehaves, I might not handle it well, and then I blame myself. For instance, when I punish him by spanking, I feel terrible inside afterwards.” [Participant 09] |
|  |  | Parenting burden (eg, time demands and physical and emotional energy depletion) | - “I wish I could be like Nezha (a Chinese mythological figure) with three heads and six arms, because I feel I’m already doing everything I possibly can. I’ve given her all my available time when she’s at home, but there are still things I can’t manage. For example, I can’t cook and play with her at the same time, right?” [Participant 04] |
|  | **Behavioral factors** | | |
|  |  | Parents’ own behavioral challenges (eg, ADHD symptoms and oppositional behavior) | - “I’m diagnosed with ADHD myself...My personality is confrontational, hyperactive, and impulsive—I have the combined type. All the typical emotional issues, including depression—I’ve got them all.” [Participant 12] |
|  |  | Parental concerns about child development (eg, social skills, academic performance, and emotion) | - “I worry that the more time he spends immersed in (animated videos), the less he’ll want to do things that require calm thinking or interact with others...I’m also concerned about his emotional issues. When he’s unhappy about something, he might just walk away without a word and completely give up on his tasks. I worry that he’ll have poor self-control when he grows up.” [Participant 09] |
| **Microsystem** | | | |
|  | **Family interactions** | | |
|  |  | Inadequate intervention in a child’s problematic behaviors (eg, procrastination, distraction, and impulsivity) | - “Take singing in the bathroom—when he sang or yelled loudly at home, I didn’t correct him right away. So at school, he thinks it’s fine to yell or sing in the bathroom...And about being disorganized—I never insisted on him keeping things organized at home, so naturally at school he’s careless with his stuff and often forgets his homework.” [Participant 02] |
|  |  | Frequent family conflicts (eg, negative emotions, communication barriers, and inconsistent parenting approaches) | - “Since three adults (me, my husband, and my mom) are raising the child together, there are lots of differing opinions and we all have short tempers, so the child endures a lot of emotional stress.” [Participant 11] |
|  | **School environment challenges** | | |
|  |  | Limited understanding from teachers regarding ADHD | - “I think teachers—from preschool through elementary and middle school—they don’t really understand this condition (ADHD). They just think the child is strange.” [Participant 03] |
| **Mesosystem** | | | |
|  | **Family-school interactions** | | |
|  |  | Frequent teacher complaints causing parental stress | - “His kindergarten teacher frequently reported that he wouldn’t follow instructions. She had to remind him about 5-6 times during one class, which was really excessive. And after each reminder, it wouldn’t even last two or three minutes before he was off-task again.” [Participant 09] |
|  |  | Difficulties managing a child’s negative reactions following criticism from teachers | - “When teachers reported issues to me, I immediately scolded him: ‘How could you do this? Why did you make this mistake again after we’ve discussed it?’ Looking back, my approach was wrong. He was already criticized at school, then came home to face more criticism from me siding with the teacher. This confrontational communication eventually led him to stop listening to me.” [Participant 08] |
|  | **Family-hospital interactions** | | |
|  |  | Inconsistent ADHD diagnostic standards causing parental confusion | - “When we later saw another doctor, they didn’t conduct an IQ test. These tests are now administered by nurses across different hospitals, and the assessment quality varies significantly between experienced and inexperienced staff.” [Participant 08] |
|  |  | Conflicts between ADHD intervention requirements and parental energy | - “In the process of raising him, I would start reading books about ADHD and sign up for online courses, but I often abandoned these efforts halfway due to work commitments and other reasons. This pattern kept repeating itself in a continuous cycle.” [Participant 14] |
| **Exosystem** | | | |
|  | **Work-family interactions** | | |
|  |  | Work schedule constraints limiting parental intervention in child’s ADHD | - “After my child turned one year, I worked full-time. Until age three, the grandmother took care of them while I rarely came home, usually caught up in my company work. I was somewhat lacking in childcare during this period.” [Participant 12] |
|  |  | Career adjustments due to a child’s ADHD condition | - “Due to my child’s ADHD, I quit my job last year. I decided to prioritize helping my child before focusing on my career, believing that career success means little if my child isn’t doing well. This decision had a significant impact on my life.” [Participant 08] |
|  | **Health care access and resources** | | |
|  |  | High financial burden of ADHD interventions (eg, high cost and long treatment period) | - “Currently, we’re doing sensory integration training and medication—all self-funded as insurance doesn’t cover these. The training is extremely expensive at nearly 300 yuan per session, 2-3 times weekly. With desensitization therapy on top of that, the financial burden is heavy.” [Participant 10] |
|  |  | Difficulties in accessing professional medical resources | - “Getting an appointment at Peking University’s 6th Hospital (China’s most authoritative hospital for child mental health) for pediatric services is incredibly difficult. The slots disappear within seconds—it’s practically impossible to get one. I haven’t succeeded even once. I wonder why these pediatric appointments are in such high demand and who manages to get them? It’s puzzling how they vanish instantly.” [Participant 03] |
|  |  | Limited personalization of available medical services | - “I have ADHD myself, and I’ve noticed many differences between my symptoms and my son’s. The biggest challenge is getting personalized treatment. Take West China Hospital in Chengdu (one of the best hospital in Southwest China) for example—there are only one or two truly respected specialists. Not everyone responds well to the same treatment approach. Even nationwide, I feel there are very few doctors who can provide truly individualized treatment.” [Participant 17] |
|  |  | Difficulties in obtaining suitable parenting information for Chinese | - “During the kindergarten stage, we want to provide individualized parenting at home. While there are many parenting books on the market, most are written by and for Westerners, with very few by Chinese authors. Given the unique Chinese educational environment, many Western parenting methods simply aren’t applicable here.” [Participant 04] |
| **Macrosystem** | | | |
|  | **Societal policy** | | |
|  |  | Insufficient supportive policies (eg, educational, financial, and medical assistance) | - “I couldn’t see significant progress happening in China over the next five to ten years. While small groups are working hard to push for change, their progress is inevitably slow. It’s not that we aren’t trying, but real change requires support from higher policy levels. Even when research institutions achieve results, without sufficient backing, it’s all talk. This situation leaves me feeling somewhat helpless.” [Participant 12] |
|  | **Societal perceptions** | | |
|  |  | Stigmatization in social contexts (eg, limited acceptance and perceived discrimination) | - “It’s hard for me to admit to other parents that my child has ADHD. I’m afraid they’ll go home and tell their children to stay away from my child, warning them ‘be careful, he might hit you.’ They’ll become defensive and prejudiced. This social stigma makes me feel powerless.” [Participant 16] - “These days, it’s not just parents of ADHD or autistic children—once you become a mother, you’re automatically downgraded to a second-class citizen. Society’s tolerance needs improvement, not just for special needs children, but for all children in general.” [Participant 11] - “We saw news about a child with ADHD whose entire class—over 40 parents—joined forces to demand their transfer to another school. If this happened to my child, it would feel terribly unfair. Yet, I can somehow understand those parents’ perspective too.” [Participant 10] |
|  |  | Impact of traditional educational values | - “My child barely passes each subject. In Beijing, this likely means they won’t get into high school. Their poor short-term memory means they’ll gradually fall further behind their peers. Yet they still have to attend school with children their age. It’s terrifying...unless they quit school entirely, but...” (sobbing) [Participant 03] - “Teachers have their own evaluation system. When they’re being assessed based on academic performance, how can they spare extra energy for children with special needs?” [Participant 17] |
| **Chronosystem** | | | |
|  | **Historical and social changes** | | |
|  |  | Exacerbation of ADHD symptoms during pandemics (eg, physical activity and communication skills) | - “His ADHD might have developed during the pandemic due to lack of exercise and my frequent business trips—sometimes I’d get stuck somewhere due to lockdowns. I wasn’t there enough for him...At first, I just thought he was being naughty, but after the pandemic, when he returned to kindergarten, he started ignoring teachers’ instructions.” [Participant 02] |
|  |  | Ongoing development of ADHD support systems in China | - “In some countries, once a child is diagnosed with ADHD, they’re provided with a service dog at school. When the dog sits quietly, the child sits too, and others don’t view them strangely. Our country still has a way to go in this regard—there’s a gap in understanding. Teachers might not even understand ADHD and try to enforce standard expectations, causing more pressure and psychological trauma for these children.” [Participant 09] - “There’s still a significant gap between us and some developed countries. Our supportive systems are not adequate yet.” [Participant 07] |
